# Unilateral salpingectomy in Sprague Dawley rats and its effect on litter size

**DOI:** 10.4102/jsava.v92i0.2101

**Published:** 2021-01-28

**Authors:** Elizabeth G. Bester, Martin Schulman, Robert M. Kirberger, Marthinus Hartman

**Affiliations:** 1 Department of Companion Animal Clinical Studies, Faculty of Veterinary Science, University of Pretoria, Onderstepoort, South Africa; 2 Department of Production Animal Studies, Faculty of Veterinary Science, University of Pretoria, Onderstepoort, South Africa

**Keywords:** salpingectomy, litter size, rat sterilisation, unilateral salpingectomy, placental weights, foetal weights

## Abstract

The study described a novel, rapidly performed, successful and safe surgical procedure in rats to achieve a reduction in the number of conceptuses. The objectives were to investigate the total foetal count and foetal health in both uterine horns after unilateral salpingectomy compared to the control group. Sixteen female Sprague-Dawley rats (*Rattus norvegicus*) were allocated to the study of which 10 rats underwent unilateral salpingectomy with six controls before all 16 were mated at 8–10 weeks of age. Gestational length was taken as 22 days, determined from the day of appearance of the copulatory plug. The female rats were terminated on day 19 or 20 of the gestational period. The foetuses in each horn were mapped and counted for comparison between the salpingectomy and control groups. The gravid uteri, individual foetal weights and placental weights were measured and compared between the two groups. This study described a novel, rapidly performed, successful and safe surgical procedure in rats. The mean number of foetuses in the salpingectomy group was significantly smaller than the control group. No significant differences in foetal and placental development were observed between the groups. These observations support future investigation of unilateral salpingectomy in other species as an alternative surgical method for population control.

## Introduction

Rats, particularly Sprague Dawley rats, are often used as experimental animals in reproductive studies (Drachman, Root & Wood 1966; Horiuchi et al. 1976; Hsu & Lai 2007; Sukov & Barth 1998). In addition, investigation of alternative methods to provide solutions for different challenges in the animal population management of various domestic and wildlife species is an active area of investigation.

Permanent sterilisation, including in captive wild felidae, may be controversial and various alternative non-surgical population control methods have been described. Reported successful non-surgical methods applied in various species include subcutaneous implantation of the gonadotropin-releasing hormone (GnRH) analogue deslorelin (for down-regulation of pituitary gonadotropin secretion) and immunocontraception using anti-zona pellucida vaccines and anti-GnRH vaccines (Bertschinger et al. 2001, 2008; Fayrer-Hosken 2008; Kirkpatrick, Lyda & Frank 2011). Surgical sterilisation options include ovariohysterectomy, uterine horn occlusion, ovariectomy, laparoscopic-assisted ovariohysterectomy, hysterectomy and salpingectomy (Hartman et al. 2015a, 2015b, 2015c).

Unilateral ovariectomy in mice, rats, pigs and dogs reportedly result in compensatory changes to the remaining contralateral ovary, including compensatory follicular development, increased ovulation rates and increased numbers of ovulated oocytes (Baker, Challoner & Burgoyne 1980; Benson, Sorrentino & Evans 1969; Bhattacharya 2013; Dailey et al. 1970; Hori et al. 2011; Ramirez & Sawyer 1974; Razi et al. 2010; Rexroad & Casida 1976; Short et al. 1968; Tsutsui et al. 2012). This increased unilateral ovulation rate continued for at least 75 weeks in mice (Biggers, Finn & McLaren 1962) and 8 weeks in hamsters (Sengupta, Chaudhuri & Bhattacharya 2013). Bhattacharya (2013) demonstrated that compensatory hypertrophy and ovulation were unaffected by unilateral ovariectomy and thus had no effect on the rate of fertilisation in mice (Bhattacharya 2013). The rat has two separate uteri, each with its own cervix (Hamilton 1947). The study by Bhattacharya (2013) demonstrated that fertilised oocytes did not cross over and implant in the uterine horn contralateral to their ovary of origin (Bhattacharya 2013). There are no studies reporting the number of conceptuses or litter sizes outcome of unilateral ovariectomy.

In rabbits, no differences were observed in either embryo quality or survival rates between intact and unilaterally ovariectomised females (Peiro et al. 2014). This suggested that unilateral ovariectomy was not associated with any physiological or hormonal imbalance leading to increased embryo loss (Peiro et al. 2014). It further suggested that fertility in various species is apparently unaffected by unilateral ovariectomy.

Similarly, investigating the ability of unilateral salpingectomy to affect litter size or influence foetal health in rats may provide a useful model supporting this as an alternative surgical method for animal population control.

## Materials and methods

### Animals

Sixteen virgin female Sprague-Dawley rats *(Rattus norvegicus)*, housed and maintained at the University of Pretoria Biomedical Research Centre, were allocated to this study, which was approved by the University’s Animal Use and Care Committee (protocol number: V117–17). Rats were randomly allocated to two groups: 10 underwent unilateral salpingectomy (salpingectomy group) and six acted as treatment controls (control group).

### Unilateral salpingectomy

The body weights of all female rats were recorded (Jadever JWA scale, Qingdao, China) on the day of surgery. Ten female rats starved from the evening before being anaesthetised using a face mask connected to a non-rebreathing circuit and by adjusting the oxygen flowmeter to approximately 4 L/min. Sevoflurane was chosen as the anaesthetic agent because of the associated advantages of fast induction and recovery and the limited effect on foetal development (Danneman & Mandrell 1997; Flecknell 2015; Fujinaga et al. 1987; Holaday et al. 1975; Kennedy et al. 1977; Mazze et al. 1985; Schaeffer 1997). The vapouriser was adjusted to approximately 8% for induction and approximately 4% for maintenance. The flowmeter was adjusted to 2–4 L/min. Anaesthetic time was recorded from the start of induction to the end of the surgery.

The anaesthetised rat was positioned in right lateral recumbency, the left dorso-lateral flank hair was removed with a clipper (Andis AGC professional clipper, Sturtevant, United States [US]), and the surgical area prepared by washing three times with 4% chlorhexidine gluconate bioscrub soap (Macmed^®^, Nottingham, United Kingdom [UK]) and then wiped three times with 0.5% chlorhexidine in 70% alcohol. A subcutaneous horizontal line block using ropivacaine (Naropin^®^) at 2 mg/kg, caudal to the last rib in the sterile prepared location was carried out before a second preparation with 0.5% chlorhexidine in 70% alcohol.

**FIGURE 1:**
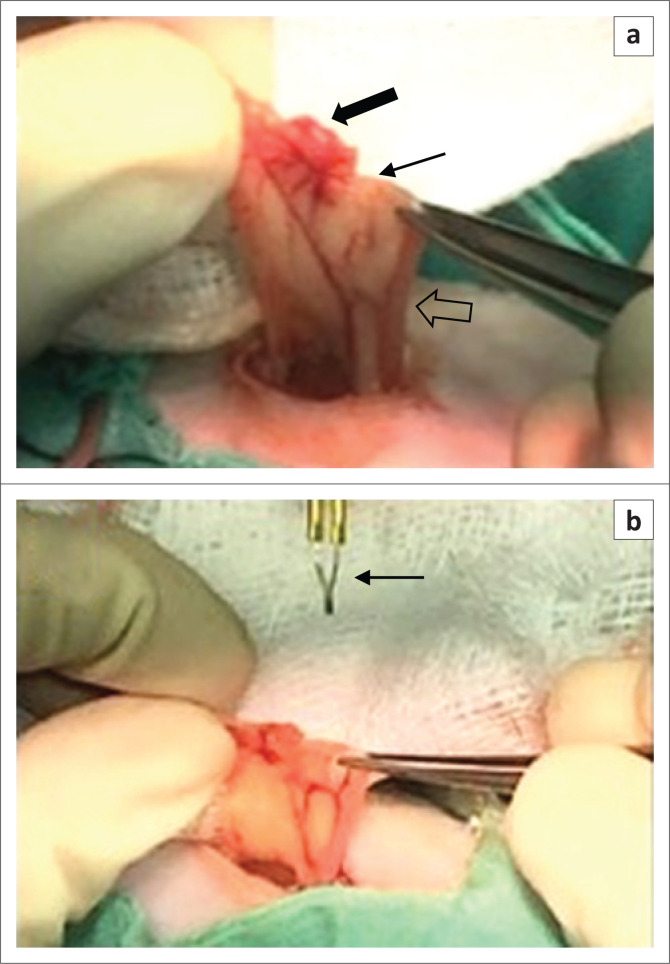
The incised ovarian bursa and isolated salpinx. (a) Isolation of the salpinx after careful incision of the ovarian bursa. Solid thick black arrow indicates ovary, thin black line arrow indicates salpinx and black arrow outline indicates uterine horn. (b) The distal portion of the isolated salpinx was resected using fine-tipped electrocautery indicated by thin black arrow.

Surgery was simultaneously performed by two surgeons on the left salpinx of all rats, one exteriorised the ovarian bursa and isolated the salpinx from the ovary, and the other resected the distal portion of the isolated salpinx with a fine tip cautery instrument (Jorgensen Laboratories Inc., Loveland Co., US). A single 20-mm long dorsolateral longitudinal incision, 20-mm ventral to the dorsal spinous processes, starting at the caudal aspect of the last rib, was made. A white fat pad was visualised through the muscle layer and a horizontal abdominal incision made approximately 5 mm long over this fat pad. The fat pad surrounding the ovarian bursa was extracted through the incision, exposing the ovarian bursa and cranial uterine horn. Once exteriorised, the ovary’s vascular supply was easily identified. The ovarian bursa was carefully opened with a fine tip cautery instrument (Jorgensen Laboratories Inc. AA; Loveland Co., US). The salpinx was cautiously isolated from the ovary with fine Bonaccolto conjunctival forceps (Figure 1). The distal portion of the salpinx was resected using fine tipped electrocautery and was submitted for histologic examination checked for haemorrhage and replaced. A simple cruciate suture using 5/0 PDS was placed in the muscular defect followed by simple continuous intradermal skin sutures using 5/0 PDS. A layer of Surgi-lock skin glue (Meridian Animal Health^TM^, UK) was applied to the incision. Surgery time from the first incision to the last skin suture was measured and recorded.

The 10 rats were allowed to recover fully from anaesthesia under supervision in individual recovery boxes. Buprenorphine hydrochloride (Temgesic^®^ Reckitt Benckiser Healthcare South Africa (Pty) Ltd. Elandsfontein, SA) at 0.1 mg/kg every 8 h was administered subcutaneously for 2 days post-operatively and thereafter as deemed necessary for pain control.

### Mating

The male rats were introduced to both the unilateral salpingectomy and the control groups at 8–10 weeks of age, approximately 10 days post-surgery for the unilateral salpingectomy, when their body weights were between 150 and 200 g. Mating was confirmed in all rats by recording the presence of copulatory plugs (Ochiogu, Uchendu & Ihedioha 2006). We initially attempted to determine mating by evaluating the presence of spermatozoa on a vaginal cytology every morning (Goldman, Murr & Cooper 2007).

### Termination and macroscopic examination

The female rats were terminated on day 19 or 20 of the gestational period. Gestational age was measured from the time of presumed conception, with day 22 regarded as the prospective day of birth (Ochiogu et al. 2006). Each rat was weighed and then humanely euthanised by overdose with isoflurane anaesthetic gas in a chamber and transabdominal ultrasonography was used to confirm that all foetal hearts had also stopped beating. The entire gravid uterus and both associated ovaries were removed. Foetal numbers were recorded and distinguished between left and right horns. The entire gravid uterus was weighed. (Jadever Snug II scale; Terre Haute, IN, US). Each uterine horn was carefully incised, exposing the lumen and allowing inspection of the implantation sites for mapping and counting. The individual foetuses and their associated placentae were removed, and their weights recorded. Individual foetal crown to rump (CR) lengths were measured using a vernier calliper (Grip Digital Vernier Calliper, Illinois, US).

### Statistical analysis

Sample size was determined on reported average litter size for Sprague-Dawley rats (Chahoud & Paumgartten 2009) and we hypothesised that unilateral salpingectomy would halve the postulated litter size. A standard deviation of 1.5, an α error probability of 0.05 and a power of 0.95 were used. A computed sample size generated a recommended sample size of four. However, because of the biological nature of the study and potential introduction of physiological stress to females during the gestational period, we decided on a sample size of 10 for the salpingectomy and six for the control groups, respectively.

Data for body weight both at the time of surgery and termination, anaesthetic time, surgery time, total number of foetuses, number of foetuses in the left uterine horn and right uterine horn, the gravid uterus weight at termination, individual foetal weight and placental weight and CR length ratio were tested for normality using the Kolmogorov-Smirnov test. Levene’s test was applied to parametric data to test for equal variances. Normally distributed data were reported as mean and standard deviation and non-parametric data as median and interquartile range.

Body weights of the female rats at the time of surgery and termination were compared using a two-tailed independent sample *T*-test. The total number of foetuses and the number of foetuses in the right horn for the two groups were compared using a two-tailed independent sample *T*-test. The gravid uterus and placental weights of the female rats in both groups were also compared using a two-tailed independent sample *T*-test. The number of foetuses in the left and right horns of rats were compared using a paired samples *T*-Test. Individual foetal weights and CR lengths were compared using an independent sample Mann-Whitney *U* Test.

A simple linear regression model determined the correlation between foetal weight and total number of foetuses, foetal weight and final female body weight and foetal weight and placental weight at termination, with foetal weight as the dependent variable. All analyses were performed with Statistical Package for the Social Sciences (SPSS) version 17 (IBM, New York, US) statistical software package. Statistical significance was defined as *P* < 0.05 in all cases.

### Ethical consideration

This study was approved by the University of Pretoria Biomedical Research Centre, Animal Use and Care Committee (protocol number: V117-17).

## Results

Unilateral salpingectomy was successfully performed in the 10 rats. Median anaesthetic time was 18 min (interquartile range [IQR] 15.8–30.0) and surgical time 9.5 min (IQR 6.8–10.8). Surgical complications included intra-operative haemorrhage in two rats from the ovarian blood vessel controlled by intra-operative fine tip cautery and dehiscence of the skin layer of the suture site in one rat 2 days post-operatively. The latter was treated as an open wound and allowed to heal by second intention. Even though copulatory plugs were present every morning, no spermatozoa were visualised upon cytology.

At the time of surgery, mean body weight of salpingectomy rats (160.4 ± 10.4 g) was significantly less than the control group rats (176.2 ± 9.1 g) (*P* = 0.009). The mean final weight of the female rats in the salpingectomy group (275.8 ± 19.2 g) was similar to that of the control group (288.8 ± 11.1 g) (*P* = 0.156).

**FIGURE 2:**
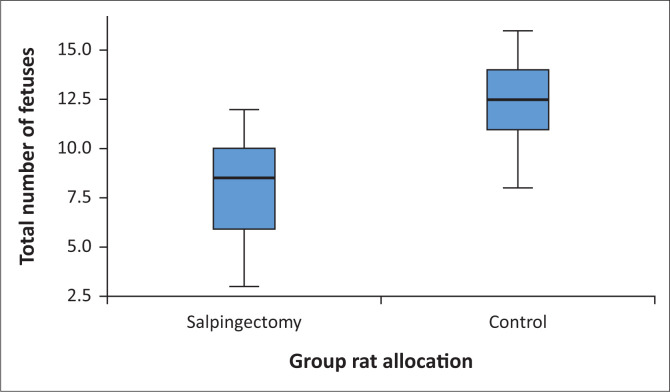
Box plot demonstarting the mean total number of foetuses at the time of termination in the salpingectomy and control groups. The blue boxes indicate the standard deviation for each individual group and the whiskers show the highest and lowest recorded values for the salpingectomy and the control groups, respectively.

The mean total number of foetuses in the salpingectomy group was significantly less (8.1 ± 2.9) compared to the control group (12.3 ± 2.7) (*P* = 0.016) (Figure 2). No foetuses were seen in the left uterine horn of the salpingectomy group compared to the control group (5.7 ± 2.3) foetuses (Table 1). The mean number of foetuses in the right horn in the salpingectomy group (8.1 ± 2.9) did not differ significantly from right horn of the control group (6.7 ± 1.2) (*P* = 0.277). In the control group, there was no difference between the left (5.7 ± 2.3) and the right horn (6.7 ± 1.2) (*P* = 0.377).

**TABLE 1:** Mean weight of females at the time of surgery and termination for the salpingectomy and control groups. Number of foetuses in the right and left uterine horns in the salpingectomy and control groups.

Variables measured	Control group		Salpingectomy group		
	Mean	SD	Mean	SD	Comparative two-tailed *P*-value
**Weight of females at surgery**	176.2	9.1	160.4	10.4	*P* = 0.009
**Weight of females at termination**	288.8	11.1	275.8	19.2	*P* = 0.156
**Uterine horn**	-	-	-	-	-
R	6.7	1.2	8.1	2.9	-
L	5.7	2.3	0	N/A	-

L, left; R, right; SD, standard deviation; NA, not applicable.

**FIGURE 3:**
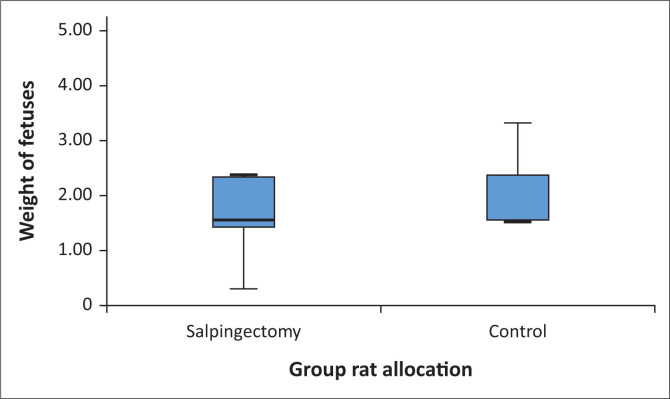
Box plot demonstrating the median individual weight (in grams) of foetuses at the time of termination in the salpingectomy and control groups. The median individual foetal weight in the salpingectomy group is 1.6 with upper and lower quartile values of 1.4 and 2.3, respectively. The median individual foetal weight in the control group is 1.6 with upper and lower quartile values of 1.5 and 2.6, respectively. The whiskers show the 5th and 95th percentiles of the values, respectively.

The mean uterine weight of the salpingectomy group (33 ± 8.5 g) was significantly less than the control group (47.6 ± 16.4 g) (*P* = 0.033). Median individual foetal weight in the salpingectomy group (1.6; IQR 1.4–2.3 g) did not significantly differ from the control group (1.6; IQR 1.5–2.6 g) (*P* = 0.428) (Figure 3). Similarly, mean placental weight of the salpingectomy group (0.6 ± 0.2 g) showed no significant difference compared to the control group (0.6 ± 0.1 g) (*P* = 0.428). There was no difference in the median CR length of the salpingectomy foetuses (23.1; IQR 20.4–28 mm) compared to the control group (24.5; IQR 23.1–37.2 mm) (*P* = 0.263).

There was a poor correlation between total number and weight of foetuses in both the control (*r* = 0.601) and salpingectomy (*r* = 0.199) groups. There was however, a stronger correlation between weight of foetuses and the final weight of female rats in the control group (*r* = 0.881) compared to the salpingectomy group (*r* = 0.571). There was a stronger correlation between weight of foetuses and the placental weight in the salpingectomy group (*r* = 0.744) compared to that for the control group (*r* = 0.446).

## Discussion

The surgical technique for unilateral salpingectomy, described in this study, was shown to be quick and effective with no major surgical complications. It was found that two surgeons optimised the surgical procedure. No treated rats showed pregnancy in the ipsilateral uterine horn.

In this study, the confirmation of mating was based on recording the presence of copulatory plugs (Ochiogu et al. 2006) and the accuracy of this confirmation with the subsequently observed gestational period was similar to that reported previously (Goldman et al. 2007).

Lower body weights of the salpingectomy rats at the time of surgery was believed to be because of the pre-operative starving of the salpingectomy rats. Khajuria, Razdan and Mahapatra (2012) demonstrated the mean surgery time for a dorsolateral incision for ovariectomy (9.7 ± 0.9 min) in rats (Khajuria et al. 2012), which is similar to our mean surgery time. The final body weight in this study did not significantly differ between groups, although the slightly heavier control group was attributed to a larger uterine weight because of the presence of more foetuses.

The total number of foetuses in the salpingectomy group was significantly less than the total number of foetuses in the control group. These foetuses in the treated rats, similar to a previously reported study did not crossover to the contralateral uterine horn (Bhattacharya 2013). Trans-cornual migration of embryos has been previously reported in dog (Tsutsui et al. 2002), and especially in pigs (Dhindsa, Dziuk & Norton 1967; Geisert, Johnson & Burghardt 2015). This was probably because of the failure of uterine spacing and overcrowding in larger litter sizes as it is generally accepted in polytocous species that foetal growth is affected by competition from the littermates (Bruce & Wellstead 1992). Our study control group demonstrated equal numbers of foetuses within the right as well as in the left uterine horns individually as comparable to the unicornual numbers in the right uterine horn seen in the salpingectomy group.

Total foetal numbers in our salpingectomy group showed a significantly smaller number compared to the control group. In the salpingectomy group, the number of conceptuses in the contralateral horn from the unilateral salpingectomy group was insignificantly greater. These conceptuses did not cross to the contralateral uterine horn side. This was attributed to the ‘double’ rat uterus, in which each horn has its own cervix allowing individual communication with the vagina unlike the female reproductive anatomy of other species (Hamilton 1947).

Gravid uterine weights were significantly lower in the salpingectomy group, compared to the control group, because of the smaller mean total number of foetuses. Importantly however, the individual foetal weights, placental weights and CR lengths of the foetuses in both groups were not significantly different. This strongly suggests that foetal development and health were not compromised by unilateral salpingectomy. The foetal weights in a study with unilateral ovariectomy in mice showed a similar insignificant difference between the groups as confirmed in our study (Dewar et al. 1984). The poor correlations observed between mean foetal number and mean weights of the females in the control rats and also between mean foetal and mean placental weights in the salpingectomy rats are difficult to explain; however, this would be beneficial as one would expect that a small number of foetuses could relay to larger foetuses, increasing the risk of potential dystocia (Catheline et al. 2006). The stronger correlation between mean weight of foetuses and the mean final weight of female rats in the control group would support the argument that bigger females would provide bigger foetuses. Neither Benson or Baker investigated the correlation between the weight of the foetuses and the total number of foetuses in rats and mice, respectively (Baker et al. 1980; Benson et al. 1969).

In conclusion, this study described a novel, rapidly performed and successful surgical method for unilateral salpingectomy in rats, which resulted in a significant decrease in litter size. Furthermore, no detrimental effect on foetal and placental development was found. These observations support further investigation of unilateral salpingectomy as a viable alternative for surgical population control in other species.
